# Novel and Modified Neutrophil Elastase Inhibitor Loaded in Topical Formulations for Psoriasis Management

**DOI:** 10.3390/pharmaceutics12040358

**Published:** 2020-04-14

**Authors:** Andreia Nunes, Joana Marto, Lídia Maria Gonçalves, Sandra Simões, Rita Félix, Andreia Ascenso, Francisca Lopes, Helena Margarida Ribeiro

**Affiliations:** 1 Faculty of Pharmacy, Universidade de Lisboa, 1649-003 Lisbon, Portugal; andreia.a.nunes@campus.ul.pt; 2 Research Institute for Medicine (iMed.ULisboa), Faculty of Pharmacy, Universidade de Lisboa, 1649-003 Lisbon, Portugal; lgoncalves@ff.ulisboa.pt (L.M.G.); ssimoes@ff.ulisboa.pt (S.S.); ritafelix@ff.ulisboa.pt (R.F.); andreiaascenso@ff.ulisboa.pt (A.A.); fclopes@ff.ulisboa.pt (F.L.)

**Keywords:** human neutrophil elastase inhibitors, emulsions, microemulsions, psoriasis, topical application

## Abstract

Human neutrophil elastase (HNE) is a serine protease that degrades matrix proteins. An excess of HNE may trigger several pathological conditions, such as psoriasis. In this work, we aimed to synthesize, characterize and formulate new HNE inhibitors with a 4-oxo-β-lactam scaffold with less toxicity, as well as therapeutic index in a psoriasis context. HNE inhibitors with 4-oxo-β-lactam scaffolds were synthesized and characterized by NMR, FTIR, melting point, mass spectrometry and elemental analysis. In vitro cytotoxicity and serine protease assays were performed. The compound with the highest cell viability (AAN-16) was selected to be incorporated in an emulsion (AAN-16 E) and in a microemulsion (AAN-16 ME). Formulations were characterized in terms of organoleptic properties, pH, rheology, droplet size distribution, in vitro drug release and in vivo psoriatic activity. All compounds were successfully synthesized according to analytical methodology, with good yields. Both formulations presented suitable physicochemical properties. AAN-16 E presented the most promising therapeutic effects in a murine model of psoriasis. Overall, new HNE inhibitors were synthesized with high and selective activity and incorporated into topical emulsions with potential to treat psoriasis.

## 1. Introduction

Psoriasis is a chronic inflammatory skin disease that affects at least 100 million individuals worldwide. It is characterized by red, scaly and erythematous plaques that can be scattered over the entire surface of the body or on a specific area, including the scalp, nails and joints. Psoriasis is considered a non-communicable disease with no cure, and it may progressively worsen with age, depending on the degree of severity [1,2,3]. The causes of psoriasis are not totally understood, but its pathogenesis is associated with genetic and epigenetic modifications that can be triggered by environmental factors [2,4]. Neutrophil chemokines, such as CXCL1, CXCL2 and CXCL8/IL-8, are usually present in psoriatic skin [5,6].

Despite the effort to find biologic drugs for the treatment of moderate to severe plaque type psoriasis based on the imunopathogenesis of the disease, the current treatment of psoriasis is mostly based on control of symptoms. Currently, phototherapy (ultraviolet light), systemic and topical therapies using corticosteroids, vitamin D analogous, retinoids and dithranol are commonly used [3,4,7]. Nevertheless, these therapies have numerous disadvantages. Therefore, it is extremely important to keep searching for new alternatives and discover new active compounds that may be able to manage this disease.

Human neutrophil elastase (HNE) is a serine protease with a single polypeptide chain that belongs to the elastase-like serine proteases subfamily, being stored and secreted from polymorphonuclear neutrophils [8,9,10,11]. Its activity depends on a catalytic triad consisting of Ser195, His57 and Asp102, which are all at the active site of the enzyme [10,12,13].

In terms of biological function, HNE has bactericidal, tissue preservative and restorative properties. Moreover, HNE is also involved in inflammation. In a normal inflammatory process, neutrophils release a high concentration of HNE to the extracellular medium, where this enzyme can modulate the immune response through degradation of pro-inflammatory cytokines, reducing the intensity of the inflammation [13,14,15]. However, if an imbalance between HNE and its endogenous inhibitors occurs during this process, the extracellular HNE in excess can degrade structural proteins of the extracellular matrix, like elastin, proteoglycan, collagen and fibronectin. Hereupon, the excess of HNE may trigger several pathological conditions and tissue injuries inducing chronic inflammatory diseases, such as psoriasis and cystic fibrosis, among others.

Accordingly, HNE is a valuable therapeutic target, and the design of new HNE inhibitors able to modulate the proteolytic activity of HNE represents a promising approach for psoriasis, where there is an excess in the production of this serine protease [12].

Under physiological conditions, endogenous HNE inhibitors tightly regulate their activity, avoiding degradation of connective tissue proteins [12,14]. These inhibitors can belong to two different families depending on their tissue location—the serpins and the chelonianins [14,16]—and can be categorized into non-covalent and covalent inhibitors, as well as reversible or irreversible inhibitors [10,17,18]. It is worthy pointing out that elafin, one of the HNE inhibitors, was the first to be isolated from the skin of psoriatic patients. This inhibitor has a second-order constant association of 5 × 10^6^ M^−1^·s^−1^ and is considered a potent reversible inhibitor of this enzyme [14,19]. Over the years, numerous HNE inhibitors such as 4-oxo-β-lactams, benzoxazinones, diazo compounds or haloketones have been developed [9,20,21]. In this context, it is necessary to consider not only the size and chemical nature of the structure, to decide the route of administration, but also the enzyme selectivity and appropriate pharmacokinetic and pharmacodynamic properties [14,18]. Synthetic HNE inhibitors need to have some peptide character to act as effective drugs, since binding of an aliphatic amino acid side chain in the S1 pocket is more favored. Notwithstanding, peptides usually present chemical instability, poor pharmacological profile and low bioavailability. These properties could be improved when low molecular weight drugs are synthesized (≤500 Da) and physicochemical properties (log *P* = 2–3, ionization, etc.), drug concentration and diffusion through the skin are optimized [18,22].

**Scheme 1:**
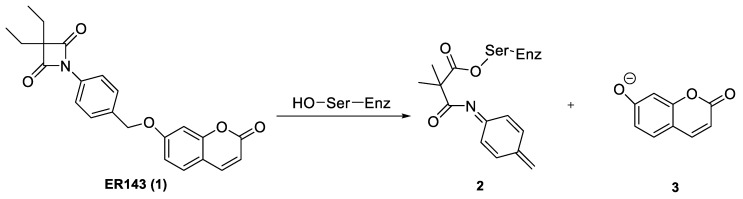
Reaction between HNE and ER143 (1).

Recent studies showed that the structure of β-lactam inhibitors allows a great improvement on the rate of serine acylation since they increase the intrinsic chemical reactivity, when compared to other compounds [10,15,23]. The synthesis of new compounds with a β-lactam structure, such as azetidine-2,4-diones, also known as 4-oxo-β-lactams, were demonstrated to be a very potent and selective class of HNE acylating agents (Scheme 1). The structure-activity relationship (SAR) studies performed by Mulchande, J. et al. [24] showed that substituents at C-3 of the four-membered ring are very important for the potency of this inhibitor. Furthermore, these studies indicated that a diethyl substitution in the 4-oxo-β-lactam scaffold, along with *N*-phenyl derivatization of this structure, selectively improved the activity against HNE [24]. Accordingly, previous studies reported by our group [24,25,26] also showed that these compounds could be quite promising regarding the inhibition of serine proteases. In particular, ER143 (1) [21] showed high potency and selectivity for HNE but it presented poor solubility and cell viability when tested in immortalized human keratinocyte (HaCaT) cells [21]. Thus, these new HNE inhibitors were synthesized as an elaboration of ER143 (1) (3,3-diethyl-1-(4-(((2-oxo-2*H*-chromen-7-yl)oxy)methyl)phenyl)-azetidine-2,4-dione), as reported in Scheme 1, previously synthesized by our research group [21], and was used as a control [10,15,23].

Topical delivery systems are used to vehiculate active substances across the skin barrier, affecting not only the drug potency, but also the acceptability by the patient [27]. There are several types of topical pharmaceutical forms, including creams, gels, ointments, pastes, etc. Emulsions are thermodynamically unstable systems composed of two immiscible liquids (water and oil) stabilized by surfactants that reduce the interfacial tension between those phases. The nature of the emulsion is influenced by the water and oil ratio, the type and geometry of the surfactant used in the formulation and the manufacturing process. Emulsions require a certain amount of energy, thus, rotor stators are commonly used in the production of emulsions allowing the dispersion of the droplets [28,29,30].

Over time, the droplets, through Brownian movement, tend to collide with each other to reduce the interfacial area. Consequently, coalescence will occur and continue until full phase separation and the minimum free energy (equilibrium) are reached. For this reason, emulsions are one of the most difficult and complex systems in the pharmaceutical field to develop and stabilize, requiring expertise knowledge and experience [29,30]. To prepare emulsions with the shelf life stability required for a pharmaceutical product (1–3 + years), the correct choice of surfactants, co-surfactants, fatty alcohols, polymers (steric stabilization) or solid particles (Pickering emulsions), among other excipients, must be made [29,31].

Microemulsions are considered to be dispersed colloidal systems consisting of two liquid phases and with a clear and translucid macroscopic aspect mainly due to their reduced droplet size. These formulations differ from emulsions in many aspects, such as macroscopic appearance, particle size, stability, formation and composition [32,33]. In addition to the components mentioned above, microemulsions further require a higher content of surfactant and a co-surfactant, usually a low molecular weight alcohol, which may compromise their safety [33,34,35]. The formation process of microemulsions is significantly easier since they form spontaneously (almost zero interfacial tension) without the need for high energy input. In practice, only gentle agitation is required [29,33,34], which makes these systems quite attractive for the industrial field.

In this work, the synthesis and full characterization of new HNE inhibitors with 4-oxo-β-lactam scaffolds and less toxicity are reported. After compound selection, two different topical forms are developed as O/W emulsions and microemulsions further characterized in terms of physicochemical properties, in vitro drug release and in vivo antipsoriatic activity.

## 2. Materials and Methods

### 2.1. Materials

#### 2.1.1. Synthesis of HNE Inhibitors

All reagents and solvents were purchased from Sigma-Aldrich (Munich, Germany), Alfa Aesar (Heysham, Lancashire, UK) and TCI (Zwijndrecht, Belgium) and used without further purification. Dichloromethane (DCM) and acetone were dried by distillation from calcium hydride and potassium carbonate, respectively.

#### 2.1.2. Formulation of HNE Inhibitor Loaded Emulsion and HNE Inhibitor Loaded Microemulsion

In this study, the AAN-16 (compound **16**) was incorporated in an O/W emulsion and in a microemulsion named “AAN-16 E” and “AAN-16 ME”, respectively.

In AAN-16 E, glycerin (Quimidroga, Portugal) was used as a humectant and methylparaben and propylparaben (Nipagin™ M Sodium and Nipasol M Sodium™, Clariant, Muttenz, Switzerland) as preservatives. In addition, hydrogenated lecithin (and) C12-16 alcohols (and) palmitic acid (Biophilic™ H MB, IFF Lucas Meyer Cosmetics, Quebec City, QC, Canada), caprylic/capric triglycerides (Tegosoft^®^ CT, Evonik, Essen, Germany), C10-18 triglycerides (Lipocire™ A SG, Gattefossé, Saint-Priest, France), isopropyl myristate (Tegosoft^®^ M, Evonik, Essen, Germany), oleic acid (Sigma-Aldrich, Munich, Germany), alpha bisabolol (Esperis, Milan, Italy), cetyl alcohol (DS produtos químicos, Cascais, Portugal) were also added to AAN-16 E. On the other hand, C21-C28 alkane (Emosmart ™ C28, Seppic ™, La Garenne-Colombes, France), a plant-based and renewable C15-19 alkane oil (Emogreen ™ L15, Seppic ™, La Garenne-Colombes, France) and caprylic/capric triglycerides (Tegosoft^®^ CT, Evonik, Essen, Germany) were added to AAN-16 ME. In addition, PEG-20 glyceryl triisostearate (Cithrol™ 10GTIS, Croda Inc, Snaith, UK) was used as surfactant and propylene glycol (Mosselman, Ghlin, Belgium) as humectant and co-surfactant, and finally, methylparaben and propylparaben (Clariant, Muttenz, Switzerland) were used as preservatives. Purified water was obtained by inverse osmosis (Millipore, Elix^®^ 3, Merck, Darmstadt, Germany).

### 2.2. Methods

#### 2.2.1. Synthesis and Characterization of HNE Inhibitors

The HNE inhibitors were synthesized according to the literature [21,24,26]. The synthesized compounds were characterized by NMR techniques, FTIR and mass spectrometry, elemental analysis and melting point as described in the Supplementary Materials.

Chemical reactions were followed by thin layer chromatography (TLC) using Merck aluminium backed sheets coated with 60 F254 silica gel and visualized under a UV lamp or revealed using iodine, potassium permanganate and vanillin. Compound purification was obtained by flash chromatography using silica gel (0.040–0.063 mm) from Merck (Darmstadt, Germany).

NMR experiments were performed on a Bruker 300 ultra-Shield (Bruker, Massachusetts, NE, USA) (^1^H 300 MHz; ^13^C 75 MHz) in chloroform-d or acetone-d_6_. All chemical shifts (δ) were quoted in ppm scale and coupling constants (J) in Hz, and multiplicity was described with the following abbreviations: s = singlet, d = doublet, t = triplet, m = multiplet.

FTIR spectra were obtained using a IRAffinity-1 (Shimadzu, Kyoto, Japan). Each compound was previously blended with potassium bromide and pressed with a hydraulic press until a pellet was formed.

Melting points (m.p.) were determined using a Bock-Monoscop (Kofler, Berlin, Germany).

The Log P was predicted using the ALOGPS 2.1 program [36].

#### 2.2.2. Biological Assays of Synthesized HNE Inhibitors

##### Enzymatic Inhibition Assays and In Vitro Cytotoxicity

Fluorometric assays for HNE inhibition activity were conducted as previously described [37]. Briefly, the assays were carried out in 200 µL assay buffer (0.1 M HEPES pH 7.5 at 25 °C) containing 20 µL of 0.17 μM HNE in assay buffer, 155 µL of assay buffer and 5 µL of each concentration of the tested inhibitors. The reaction was initiated by the addition of 20 μL of fluorogenic substrate at 200 µM (MeO-Suc-Ala-Ala-Pro-Val-AMC), and the inhibition activity was monitored for 30 min at 25 °C on a microplate reader (FLUOstar Omega; BMG Labtech, Baden-Württemberg, Germany) (excitation wavelength 380 nm and emission 460 nm). Inhibitor stock solutions were prepared in DMSO. Controls were performed as follows: (1) enzyme; (2) substrate; (3) enzyme with DMSO and (4) positive control with Sivelestat sodium salt hydrate. Assays were performed in triplicate and presented as log of inhibitor concentrations versus the percentage of activity. IC_50_ values were determined by non-linear regression analysis in GraphPad PRISM^®^ 5 software.

Compounds **1**, **16** and **17** were evaluated for their ability to inhibit other human serine proteases, namely trypsin, chymotrypsin, urokinase and kallikrein in 200 μL reaction volumes at 25 °C, as follows: 0.05 M Tris-HCl and 0.138 M NaCl at pH 8.0, 30 or 2 nM human pancreas of each protease and kallikrein respectively, and 50 or 100 μM substrate (Z-Gly-Gly-Arg-AMC.HCl for trypsine; Suc-Ala-Ala-Pro-Phe-7-amino-4-methylcoumarin for chymotrypsin; Z-Gly-Gly-Arg-AMC.HCl for urokinase; H-Pro-Phe-Arg-AMC acetate salt for kallikrein, from Bachem, Switzerland).

For all serine proteases, the activity was measured at excitation and emission wavelengths of 360 nm and 460 nm, respectively, in a microplate reader (FLUOstar Omega, BMG Labtech, Germany). The IC_50_ was determined by non-linear regression as previously published [37].

The cell viability was evaluated with the endpoint MTT (3-(4,5-dimethyl-2-thiazolyl)-2,5-diphenyl- 2H-tetrazolium bromide, Sigma Aldrich) assay performed on a human keratinocyte cell line, HaCaT (Cell line Service GmbH, Eppelheim, Germany) [38]. These cells were seeded in sterile flat bottom 96 well tissue culture plates in RPMI culture medium, supplemented with 10% FBS, 100 units of penicillin G (sodium salt), 100 μg of streptomycin sulfate and 2 mM L-glutamine and incubated at 37 °C and 5% CO_2_.

Cell viability was assessed after 24 h of incubation of HaCaT cells with 50 µM concentration of each sample. DMSO diluted in culture medium was the negative control, whereas sodium dodecyl sulfate at 1.0 mg/mL was the positive one. After exposure, the culture medium was replaced by medium containing 0.5 mg/mL MTT. The cells were further incubated for 3 h. After removing the media, the intracellular formazan crystals were solubilized and extracted with DMSO. After 15 min at room temperature, the absorbance was measured at 570 nm in a microplate reader (FLUOstar Omega). The cell viability (%) compared to control cells was calculated by [Absorbance] sample/[Absorbance]control × 100, culture medium with the same amount of DMSO as the samples used as control.

#### 2.2.3. HNE Inhibitor Topical Formulations and Characterization Studies

##### Solubility Studies

According to biological studies of synthesized HNE inhibitors, the selected compound to incorporate in final pharmaceutical formulations was compound **16** *(2-((4-(3,3-diethyl-2,4-dioxoazetidin-1-yl)benzyl)thio)-N-(3-((7nitrobenzo[c][1,2,5]oxadiazol-4-yl)amino)propyl)benzo[d]oxazole-5-carboxamide)*. To perform the solubility studies, the oily phases of AAN-16 ME and AANN-16 E were both saturated with 1 mg of compound 16 under stirring at room temperature. The solubility was measured by a fluorescence method (excitation 380 nm; emission 460 nm) at 25 °C on a microplate reader (FLUOstar Omega, BMG Labtech GmbH, Germany).

##### Preparation of HNE Inhibitor Loaded Emulsion and HNE Inhibitor Loaded Emulsion Microemulsion

The AAN-16 E was prepared by a hot method (80 °C), adding the oily phase previously melted, where the compound **16** was dissolved, to the aqueous phase under stirring using an Ultra Turrax^®^ (IKA^®^ T25 Digital, Staufen, Germany) at 7000 rpm for 1 min. On the other hand, the AAN-16 ME was prepared by dissolving the compound **16** in the oily phase with subsequent addition of the aqueous phase under stirring with a glass rod at room temperature. All formulations were carried out using a digital scale (VWR, Portugal) and their composition is shown in Table 1.

The composition of both emulsions and microemulsions was selected by taking previous studies performed by our group into account (data not shown). In those studies, several topical delivery systems (emulsions and microemulsions) were developed, optimized and characterized. Different humectants and preservatives were studied as well as the influence of different concentrations of cetyl alcohol on the final properties of the O/W emulsions. Oils containing different carbon chains were incorporated into microemulsions to mimic the skin lipid layer. At the end, the formulations with the most suitable physicochemical and sensorial properties and higher stability were chosen.

##### Characterization Studies of HNE Inhibitor Loaded Emulsion and HNE Inhibitor Loaded Microemulsion

The pH values were determined at room temperature using a potentiometer (Mettler Toledo, Columbus, OH, USA) 24 h after the production of each formulation.

To test the physical stability of AAN-16 E, an accelerated stability test based on centrifugation (Heraeus Sepatech, Hanau, Germany) for 10 min at 3000 rpm was used.

The AAN-16 ME physical stability was evaluated by observing the macroscopic appearance (translucent or opaque) of each formulation immediately after preparation.

The rheological characteristics of the formulations were examined at high shear rates using a continuous shear technique and oscillation technique in the viscoelastic region. These experiments were performed with a controlled stress Kinexus Lab+ Rheometer (Malvern, UK) using cone geometry (truncated cone angle 4° and radius 40 mm). All measurements were performed at room temperature. The shear rate method was carried out using a destructive measurement, where the shear stress of each formulation was obtained by increasing the shear rate from 0.1 s^−1^ to 100 s^−1^ and using 8 samples per decade. First, in the oscillatory method, an amplitude sweep test was performed where the shear strain ranged between 0.01 and 100 Hz, and the frequency was set at 1 Hz. Then, a frequency sweep test was performed with a shear strain of 0.1 and 5% for AAN-16 E and AAN-16 ME respectively, and a frequency range between 0.01 and 10 Hz. Ten samples per decade were evaluated in each method.

The mean droplet sizes of emulsions and microemulsions were determined using Malvern Mastersizer (with Hydro 2000S module) and Malvern Zetasizer Nano (Kassel, Germany), respectively.

To visualize the internal structure of AAN-16 E, an optical fluorescence microscope with video camera (Bresser, Rhede, Germany) was used.

##### In Vitro Release Studies

In vitro release studies were performed using vertical Franz diffusion cells (receptor volume: 4 mL, release area: 1 cm^2^) with synthetic membranes (Tuffryn^®^ 0.45 µm, hydrophilic polysulfone membrane filter; Pall Corporation (East Hills, NY, USA)) and silicone membranes (Sil-Tec, reff 500-2, 0.002”; Technical Products Inc. of Georgia (Lawrenceville, GA, USA). The samples were evenly applied (0.2 to 0.4 g of formulation correspondent to 0.02 to 0.04 mg of compound (**16**) on the surface of the membrane in the donor compartment, and sealed with Parafilm^®^ (Bemis Company, Inc, Neenah, WI, USA) to prevent water evaporation. Water:ethanol (50:50 *w/w*) with 2.5% of PEG-40 Hydrogenated Castor Oil was used as a receptor phase (approx. solubility: 0.39 mg/mL). The study was performed for 6 h at 32–37 °C under stirring, and each sample was collected (200 µL) with an interval of one hour. The amount of compound **16** released was analyzed as previously described on a Fluorescence Microplate Reader FLUOstar Omega (BMG Labtech GmbH, Germany), and the data was expressed as the cumulative released drug amount as a function of time. The data obtained was computed using DDSolver [39] (Excel-plugin module) and fitted to different kinetic models, such as: zero order, first order, the Higuchi model and the Korsmeyer–Peppas model [40,41].

##### In Vivo Antipsoriatic Activity

The experiments were conducted using female BALB/c mice at 6 weeks of age provided by Charles River Laboratories (Écully, France). Animals were housed in polypropylene cages at ambient temperature (20–24 °C), relative humidity (55 ± 5%), 12 h’ light/dark cycle and given standard diet and water *ad libitum*. All animal experiments were performed according to the animal welfare organ of the Faculty of Pharmacy, University Lisbon, approved by the competent national authority (Direção-Geral de Alimentação e Veterinária—DGAV) and in accordance with the EU Directive (2010/63/EU), the Portuguese laws (DL 113/2013, 2880/2015, 260/2016 and 1/2019) and all relevant legislation.

The in vivo studies regarding the imiquimod (IMQ)-induced psoriasis-like inflammation model were performed according to Pivetta et al. [42]. The psoriasis-inflammation evaluation criteria were assessed, based on the clinical Psoriasis Area and Severity Index (PASI), as previously described [42]. Erythema, scaling and thickening were scored from 0 to 4 (0: none; 1: slight; 2: moderate; 3: marked; 4: very marked). In the first day, all mice were shaved in the dorsum and kept in individual cages throughout the experiment. Animals were randomly selected, and five groups of animals were tested:

- Group 1 (n = 5) was treated with 60 mg of Dermovate^®^ (GSK, Algés, Portugal) (containing 0.05% clobetasol propionate) as a positive control of inflammation inhibition;
- Group 2 (n = 4) was treated with 60 mg of emulsion placebo;
- Group 3 (n = 4) was treated with 60 mg of microemulsion placebo;
- Group 4 (n = 5) was treated with 60 mg of AAN-16 E;
- Group 5 (n = 5) was treated with 60 mg of AAN-16 ME;
- Group 6 (n = 2) was used for control of non-induced and non-treated skin.

All tested formulations were spread over the mice back skin with a spatula in an average amount of 26.7 mg/cm^2^. The two placebo formulations were used as control, since they present the same composition of AAN-16 E and AAN-16 ME, without any active compound. Five hours after the treatment, 60 mg of Aldara^®^ cream (Meda AB, Solna, Sweden) containing 5% of imiquimod was applied on the back skin. This treatment lasted for 5 consecutive days. Apart from the 1st day, the animals were always subjected to daily visual inspection and measurement of body weight and skin thickness, which was measured in the application zone using a digital calliper (Fisherbrand, Leicestershire, UK). On the 6th day, the mice were sacrificed, and their dorsal skin exposed to the test compounds was preserved in a 10% formalin solution and sent for histopathological analysis. The psoriasis-inflammation evaluation criteria were assessed according to the clinical Psoriasis Area and Severity Index (PASI) as previously described [42]. Erythema, scaling and thickening were scored from 0 to 4 (0: none; 1: slight; 2: moderate; 3: marked; 4: very marked).

Formalin-fixed tissues were trimmed into longitudinal sections. Samples were then paraffin-embedded, sectioned at 4 µm and stained with heamtoxylin and eosin. Lesions were evaluated by a pathologist blinded to experimental groups. Measurements were performed in slides digitally scanned in the NanoZoomerSQ with NDP.view2 software (Hamamatsu, Japan) corresponding to the mean value obtained from 7 to 12 different points for epidermis and dermis layers.

#### 2.2.4. Statistical Analysis

One-way analysis of variance (ANOVA) and the Tukey–Kramer post-hoc multiple comparison test was used to identify the significant differences between the groups, and were performed using GraphPad PRISM^®^ 5 software. An alpha error of 5% was chosen to set the significance level. Results were expressed as mean ± standard deviation.

## 3. Results and Discussion

### 3.1. Synthesis of 4-oxo-β-Lactams

**Scheme 2:**
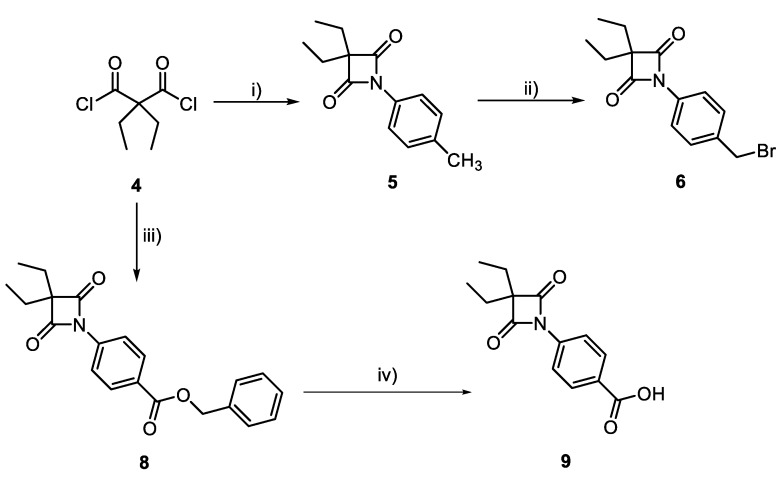
Synthesis of compound **6, 8 and 9**. Reagents and conditions: (i) *p*-toluidine, NEt_3_, DCM, r.t; (ii) NBS, benzoyl peroxide, ACN, reflux; (iii) Intermediate **7**, NEt_3_, DCM, r.t; (iv) H_2_, Pd/C, MeOH.

4-oxo-β-lactams, and in particular **ER143 (1)**, showed high potency and selectivity for HNE. However, they presented some toxicity when tested in HaCaT cells. To improve efficacy and decrease cytotoxicity, we synthesized additional analogues of **ER143 (1)** with varied physicochemical properties. As depicted in Scheme 2, the 4-oxo-β-lactam core was synthesized by reaction of diethylmalonyl dichloride with an appropriate aniline. The target compound **6** was prepared through radical bromination of intermediate **5** using NBS and benzoyl peroxide. Compound **8** was synthesized by a cyclization reaction between aniline **7** and diethylmalonyl dichloride, and subsequently, debenzylated using a palladium and hydrogen atmosphere to yield the carboxylic acid derivative compound **9**.

**Scheme 3:**
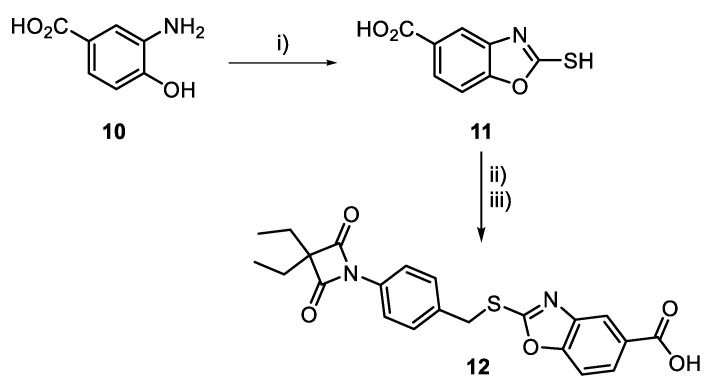
Synthesis of compound **12.** Reagent and conditions: (i) KS_2_COEt, EtOH:H_2_O (6:1), reflux; (ii) K_2_CO_3_, acetone, 20min.; (iii) Intermediate **6**, acetone, reflux.

To improve solubility of compound **6**, derivative **12** was synthesized as follows (Scheme 3). The carboxylic acid derivative **12** was prepared as depicted in Scheme 3, starting with the cyclization reaction between 3-amino-4-hydroxybenzoic acid **10** and potassium ethyl xanthate to form the benzoxazole **11**, which then reacted with compound **6** in the presence of potassium carbonate to obtain the target compound **12**.

**Scheme 4:**
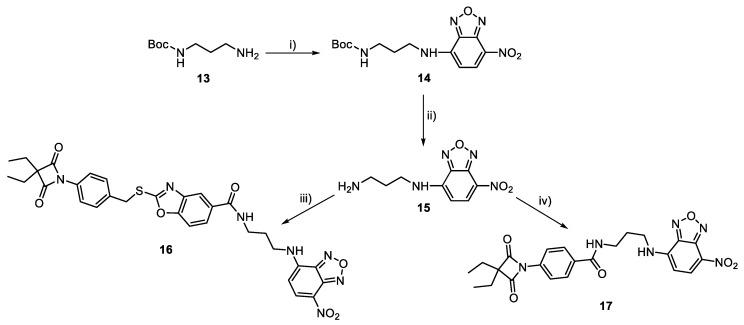
Synthesis of compound **16** and **17**. Reagents and conditions: (i) NBD-Cl, MeOH, r.t; (ii) TFA; (iii) Compound **12**, TBTU, DIPEA, DCM, 0 °C; (iv) Compound **9**, TBTU, DIPEA, DCM, 0 °C.

To allow identification and quantification of 4-oxo-β-lactams in biological assays, a synthetic scheme was devised to incorporate a fluorophore in inhibitors **9** and **12** (Scheme 4). First, intermediate **14** was synthesized by reacting amine **13** with NBD-Cl. Removal of the Boc protecting group with trifluoroacetic acid in dry dichloromethane yielded intermediate **15**, which was then converted to compounds **16** and **17**, by an amide coupling reaction with **12** and **9**, respectively, in the presence of TBTU and DIPEA. Compounds **6**, **8**, **9**, **12**, **16** and **17** were fully characterized by ^1^H and ^13^C-NMR and mass spectrometry.

**ER143 (1)** was also synthesized as described elsewhere [21] to be used as a control.

### 3.2. Biological Assays of Synthesized HNE Inhibitors

#### Enzymatic Inhibition Assays and In Vitro Cytotoxicity

To understand the activity of synthesized compounds against HNE, enzymatic inhibition assays were assessed as shown in Table 2.

By analyzing these data, 4-oxo-β-lactam was revealed to be essential for this activity, as IC_50_ values were obtained at a sub-nanomolar range, except for compound **9**. Thus, it was possible to confirm the importance of the β-lactam ring in the structure of these molecules, allowing a great improvement on the rate of serine acylation, as confirmed in [26].

From these results, none of the synthesized compounds with the oxo-β-lactam moiety could be excluded, and thus a cell viability assay was carried out with HaCat cells to choose the compounds.

**Figure 1:**
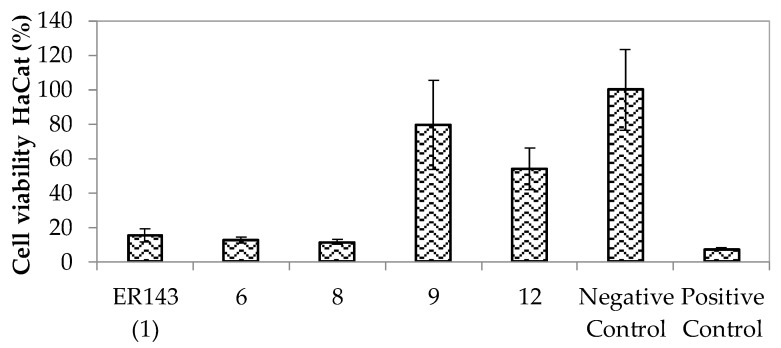
Cell viability assay at 50 µM concentration of compounds **ER143 (1)**, **6**, **8**, **9** and **12** in HaCaT cells for 24 h of exposition time.

All compounds presented lower cell viability than the negative control (DMSO), except compounds **9** and **12** (>50%) (Figure 1). The lower cell viability of compound **6** might be due to the presence of a bromine atom that is described as potentially cytotoxic, causing a decrease in cell viability [43]. Compound **8** showed the lowest cell viability among all compounds. The only difference with compound **9** was the presence of an ester group which improved the cytotoxicity.

Due to optimal inhibition activity against HNE and good cell viability of compounds **9** and **12**, fluorescent derivatives were also synthesized (compounds **17** and **16**, respectively) for the posterior drug assay.

These new compounds also showed enzymatic activity against HNE at the sub-nanomolar range (Table 3). It is also important to notice that compound **17** showed a higher activity than the parent compound **9**.

Since these new derivatives presented very similar activities, further studies of specificity were performed to select the best compound. In this study (Table 3), different enzymes were used, such as: trypsin, chymotrypsin, urokinase and kallikrein.

All the enzymes tested belong to the serine protease group, thus allowing evaluation if the compounds act only on HNE or other enzymes of the same group. The results showed that the tested compounds were specific for enzyme inhibition at a higher range like the **control ER143 (1)**. In other words, due to the high potency of these compounds, only a small amount was required to reveal activity against HNE (Table 3) which was not sufficient to act on other enzymes of the same class.

**Figure 2:**
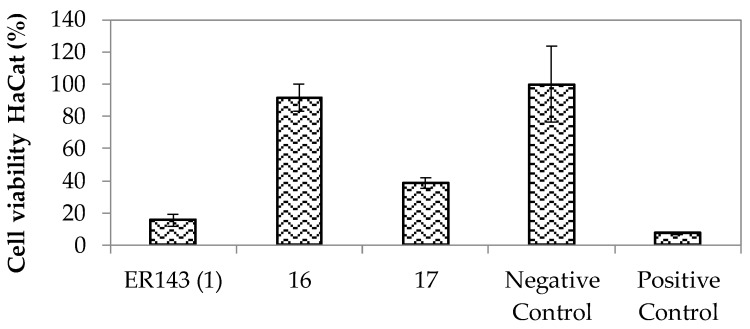
Cell viability assay at 50 µM concentration of fluorescent compounds **ER143 (1)**, **16** and **17** in HaCaT cells for 24 h of exposition time.

Compound **17** was more cytotoxic (38% of cell viability) than compound **9** (80% of cell viability), and both had higher cell viability than compound **ER143 (1)** (15% of cell viability) (Figure 2). Nevertheless, compound **16** showed a cell viability (%) comparable to the negative control. Thus, this compound was selected to be incorporated into the topical formulations.

### 3.3. HNE Inhibitor Loaded Emulsion and HNE Inhibitor Loaded Microemulsion

#### 3.3.1. Solubility Studies

According to previous results, compound **16** (AAN-16) was selected to be incorporated in an O/W emulsion (AAN-16 E) and in a microemulsion (AAN-16 ME).

The results showed that 0.1 mg/g of compound **16** was the maximum drug concentration solubilized in both oil blends, composed by caprylic/capric triglycerides, C15-19 alkane and C21-C28 alkane oils for AAN-16 ME and caprylic/capric triglycerides and isopropyl myristate for AANN-16 E. Therefore, these formulations were prepared at the same concentration of active compound (0.1 mg/g of formulation).

These final formulations were fully characterized as described below.

#### 3.3.2. Characterization Studies of AAN-16 Loaded Emulsion and AAN-16 Loaded Microemulsion

AAN-16 E revealed a yellow and opaque appearance with some consistency unlike the AAN-16 ME corresponding to a clear and homogenous formulation with liquid appearance.

AAN-16 E and AAN-16 ME presented a pH of 6.7 and 7.5, respectively. These values were appropriate for topical application, respecting the physiological pH according to the literature [44,45]. Comparing with the pH obtained in placebos, there were no significant changes.

The rheological characterization was performed by continuous shear and oscillation experiments. AAN-16 E had a much higher viscosity (182.5 Pa.s) than AAN-16 ME (0.05 Pa.s) at the same shear rate (at 0.1 s^−1^), as supposed for these systems. These values remained constant without any significant differences, when compared with placebos for both formulations.

In addition, AAN-16 ME was a Newtonian fluid in which viscosity does not change when a shear rate is applied, whereas AAN-16 E was a shear-thinning fluid characterized by a viscosity decrease with increasing shear rate owing to the orientation of the molecules in the structure [46,47]. These results were in agreement with placebo studies that others had reported [29,34,48,49,50,51].

**Figure 3:**
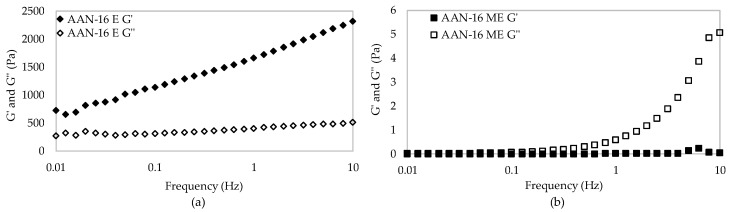
Oscillatory results of (**a**) AAN-16 E and (**b**) AAN-16 ME.

Figure 3 represents the oscillatory frequency study, in which the system response is measured as a function of frequency with constant shear strain. From these results, it is possible to obtain the storage modulus (G’) (elastic component) and the loss modulus (G’’) (viscous component) in which the energy lost is reflected [52].

In this case, the values of the elastic modulus of AAN-16 E overcame the values of the viscosity modulus (G’ > G’’) and the loss tangent values were lower than 1 [52], indicating a solid-like behavior with a strong network. In this structure type, the molecules are more bound (e.g., by Van der Walls interactions) and organized than in liquids, but less than in solids. On the contrary, the elastic modulus of AAN-16 ME was lower than the viscous modulus (G’ < G’’) and the loss tangent values were lower than 1, thus presenting a liquid-like structure with a weak and easily disturbed network. As a consequence, these formulations presented a suitable spreadability.

The d(50) for AAN-16 E was 23.52 µm with a span of 5.028, while AAN-16 ME presented a d(50) of 9.92 nm with a Pdi of 0.834. The droplet size obtained here was also concordant with the literature related to these pharmaceutical forms [29,34,51]. Although AAN-16 ME had droplets in the nano range, the Pdi value was high, indicating heterogeneity of droplet size within AAN-16 ME, or, probably a bicontinuous system. Microemulsions typically exist in the form of micellar and bicontinuous structures. However, it is very complex and difficult to characterize these systems at the molecular level.

**Figure 4:**
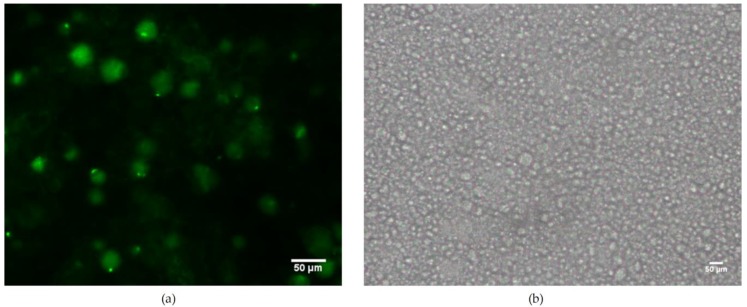
The inside structure of AAN-16 E (**a**) and emulsions’ placebo (**b**) using a fluorescence microscope.

Fluorescence microscopy was used to visualize the internal structure of these formulations with compound **16** (Figure 4).

It was only possible to observe the emulsion (E) droplets due to the respective size. In the AAN-16 E, it was possible to observe the drug both dispersed and dissolved in the formulation, as shown in Figure 4. Thus, the formulation droplets were fluorescent owing to this labeled compound.

#### 3.3.3. In Vitro Release Studies

**Figure 5:**
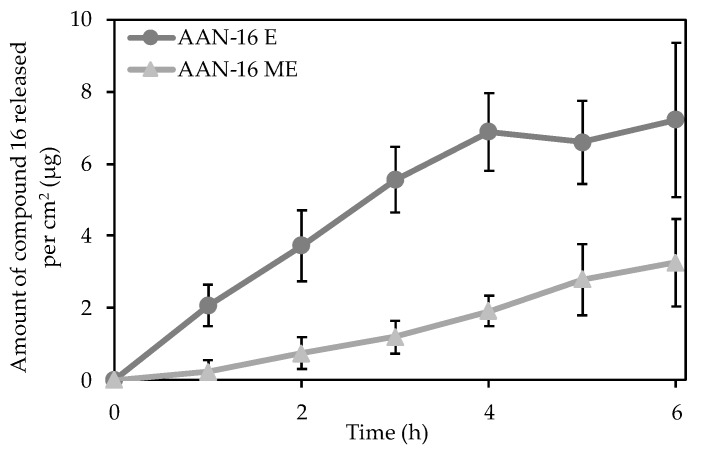
Drug release studies of AAN-16 E and AAN-16 ME using a synthetic membrane (Tuffryn^®^). Results expressed as average ± SD, n = 6.

In vitro AAN-16 release studies were performed in both final formulations using Franz Cells with a synthetic membrane (Tuffryn^®^) (Figure 5).

After 6 h, the amount of the compound **16** released was different, i.e., 7.2 ± 2.1 μg/cm^2^ and 3.3 ± 1.2 μg/cm^2^ for AAN-16 E and AAN-16 ME, respectively. The emulsion released a higher amount of drug than the microemulsion, despite the higher bulk viscosity of the AAN-16 E. In fact, the structure of vehicles plays an important role in the release of the active compound [53].

Regarding drug properties, compound **16** is mainly lipophilic (log P (predicted) = 4.68), uncharged and with a high molecular weight (M = 643.68 g.mol^−1^), thus its diffusion into the receptor aqueous phase could be compromised.

AAN-16 E and AAN-16 ME present a structure type with aqueous and occlusive lipidic phases. It was further noted that AAN-16 was dissolved in the oily phase of the microemulsion, while it was both dispersed and dissolved in the emulsion.

AAN-16 E is an O/W emulsion, where the drug is incorporated in the internal phase of the formulation. To reach the membrane and the receptor phase, the compound needs to pass through the structure of the external aqueous phase.

The diffusivity of compound **16** in the formulations was an inverse function of the surfactant concentration. In addition, several studies [54,55,56] indicate that drug flow is inversely proportional to the amount of surfactant.

Diffusion was the drug release mechanism for both formulations, where the Higuchi (R^2^_ajusted_ = 0.934 ± 0.029) and First order models (R^2^_ajusted_ = 0.784 ± 0.069) could be applied for AAN-16 E and AAN-16 ME, respectively. Finally, no drug was detected in the receptor phase when a silicone membrane was used.

The use of synthetic membranes is useful in quality assurance, but it does not represent how a formulation will behave once applied on skin. Many drugs developed for the topical treatment of skin disease are poorly water-soluble and difficult to formulate. Thus, certain strategies can be used. For example, AAN 16 was composed by permeation enhancers (isopropyl myristate, propylene glycol and oleic acid) and 6% emulsifiers (hydrogenated lecithin (and) C12-16 alcohols (and) palmitic acid), which may affect the solubility and/or interact with the membrane used [34,53,57]. Consequently, AAN-16 E exhibited higher drug release over time when compared to AAN-16 ME formulated with 40% of a non-ionic surfactant (PEG-20 glyceryl triisostearate).

#### 3.3.4. In Vivo Antipsoriatic Activity

**Figure 6:**
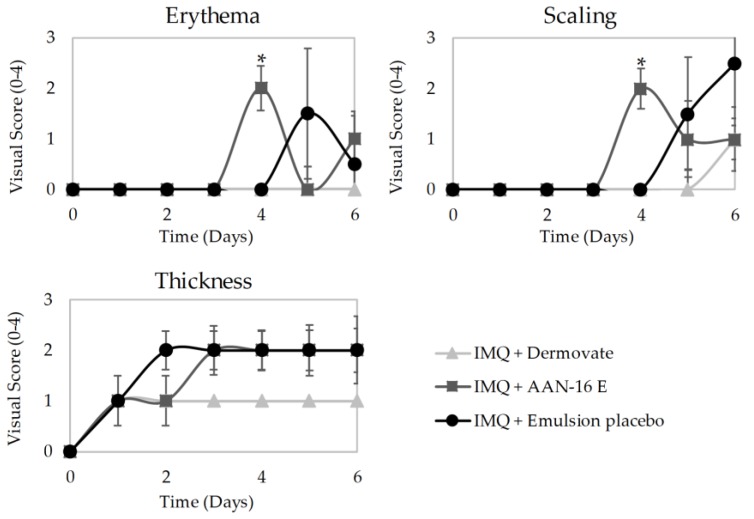
Results of the in vivo antipsoriatic activity: erythema, scaling and skin thickness of mice challenged with imiquimod (IMQ) and treated with each formulation (Dermovate^®^, AAN-16 E and emulsion placebo). Score 0–4 (none to marked). Results expressed as average ± SD.

The imiquimod (IMQ)-induced psoriasis-like inflammation model was used in this study to evaluate the antipsoriatic activity of AAN-16 E and AAN-16 ME topically applied. In this model, *stratum corneum* disturbance by imiquimod dramatically promotes the cutaneous drug absorption [58]. After treatment with test compounds and commercial corticosteroid control, several parameters were assessed as erythema, scaling, and skin thickness (Figure 6).

Mice treated with AAN-16 E and placebo obtained similar and higher scores, for all parameters in opposition to the results of the positive control group (treated with Dermovate^®^ containing clobetasol propionate). Unexpectedly, mice exposed to AAN-16 ME had to be sacrificed within 5 days of application due to the presence of physiological and behavioral signs of distress (humane endpoints). In addition, it was not possible to verify any significant difference between microemulsions’ placebo and AAN-16 ME.

IMQ-induced psoriasis-like model is a commonly used model to test antipsoriatic drugs. In BALB/c mice, IMQ topical application induces psoriasis-like dermatitis mediated via the IL-23/IL-17 axis [59]. However, in mice, this model causes not only plaque psoriasis, but also systemic disease comprising more severe symptoms, like anorexia, malaise, and pain [60]. These severe systemic features are usually only observed in non-treated animals in contrast to animals treated with antipsoriatic drugs, like clobetasol propionate cream (Dermovate^®^—Figure 6), which show amelioration in the development of IMQ-induced psoriasis-like signs. In this study, we treated mice with ME-based formulations containing approximately 40% (*w/w*) of PEG-20 glyceryl triisostearate. This compound is considered safe for cosmetic use, being favorably used as a penetration enhancer [61]. Surfactants have effects on the permeability of several biological membranes including skin mainly due to their potential to solubilize lipids within the stratum corneum. Some authors tested the cytotoxicity and inflammation potency of several surfactants on reconstructed human epidermis tissues, and concluded that PEG ethers appeared to be more toxic than PEG esters. The results also revealed the mildness of polyoxyethylene sorbitan esters independently of their alkyl chain length [62]. Frequently, the cleansing and leave-on products contain up to 20% and 5% PEG-20 glyceryl triisostearate, respectively [61]. Considering the results obtained in this work, we are convinced that the exacerbated systemic effects observed in mice treated with ME-based formulations are related to the enhanced IMQ transdermal absorption and not to the in vivo toxicity of ME-based formulation per se.

**Figure 7:**
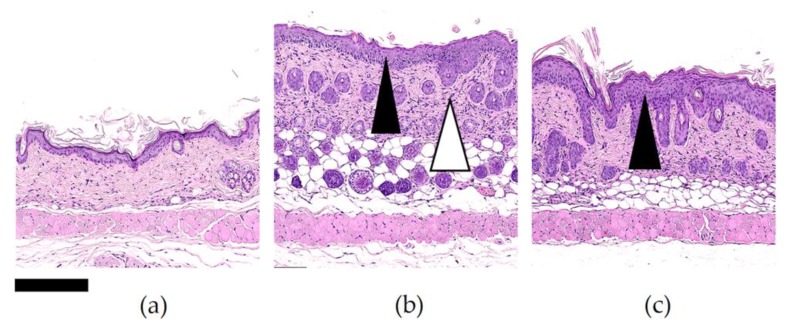
Representative microphotographs of skin (dorsum) of mice previously (**a**) treated with Dermovate^®^; (**b**) emulsion placebo; (**c**) AAN-16 E. Changes were characterized by epidermal hyperplasia (black arrowhead), and focally extensive inflammation expanding the dermis (white arrowhead). Hematoxylin and eosin stain. Original magnification, 10× (bar, 250 µm).

Figure 7 shows the histopathology results of mice skin previously exposed to imiquimod, and thereafter, to Dermovate^®^, emulsion placebo and AAN-16 E.

Histopathological changes were evaluated by means of epidermal and dermal thickness. Through the present study, it could be observed (Figure 7b,c) that the skin changes were characterized by diffuse epidermal hyperplasia resulting in increased thickness (73.91 µm and 63.48 µm for AAN-16 E and emulsion placebo, respectively) of the Malpighian layer (highlighted with a black arrow, Figure 7c,d), which is composed of *stratum basale* and *stratum spinosum*. A focally extensive minimal to mild inflammation expanding the dermis (237.13 µm and 204.79 µm for AAN-16 E and emulsion placebo, respectively) was observed promoting a diffuse thickening (highlighted with a white arrow, Figure 7). In this inflammation model, the infiltrates corresponded to a mixed cell population with a marked mononuclear-cell component. It is important to notice that the edema was minimal, and no changes were found in the untreated animals (data not show).

Therefore, the results are in accordance with the literature [42,59,63,64], where the application of IMQ causes epidermal hyperplasia resulting in increased thickness. Comparing the visual score of the emulsion placebo and AAN-16 E it is not possible to find a significant difference between these two groups. In other studies, using the same animal model, higher scores were obtained for the different parameters evaluated when placebo formulations were tested (negative control groups) [42,59,64,65]. In this study, even if it is not possible to distinguish between the AAN-16 E and the placebo results, both by visual score or by histology analysis, the results do not show high severity on skin lesions if compared to the data presented in the literature. Further studies are required to clarify the role of the placebo emulsion and its components on the inflammation control.

## 4. Conclusions

From the results presented in this work, we can conclude that these new synthesized inhibitors showed high in vitro activity and selectivity against HNE, constituting promising scaffolds. According to biological assays, only two compounds were selected (**9** and **12**), to synthesize new ones (**16** and **17**) with fluorescence labeling. As compound 16 presented higher cell viability, it was chosen to be further incorporated into topical emulsion and microemulsion formulations (AAN-16 E and AAN-16 ME, respectively). The in vitro AAN-16 release studies showed a great dependence on the formulation type. In particular, a higher release rate was obtained for emulsion compared to microemulsion.

Finally, according to in vivo antipsoriatic activity data, no significant differences between the placebo and AAN-16 E were observed. Moreover, results obtained for the tested formulations were not equivalent or superior to those of the reference formulation (Dermovate^®^), indicating that the therapeutic effect was not detected in this model. Consequently, future in vivo studies will provide a clarification of the mechanism of action of this compound among others within this scaffold.

## Figures and Tables

**Table 1:** Qualitative and quantitative composition of formulations.

Disperse Phase	Ingredients (INCI)	Formulations (%, *w/w*)	
		AAN-16 E	AAN-16 ME
Aqueous phase	Water	72.79	5.97
	Hydrogenated lecithin (and) C12-16 alcohols (and) palmitic acid	6.00	-
	Glycerin	5.00	-
	PEG-20 glyceryl triisostearate	-	40.27
	Propylene glycol	-	5.97
	Methylparaben	0.18	0.11
	Propylparaben	0.02	0.01
Oily Phase	Caprylic/capric triglycerides	2.50	15.90
	C15-19 alkane	-	15.90
	C21-C28 alkane	-	15.90
	C10–18 triglycerides	4.00	-
	Isopropyl myristate	5.00	-
	Oleic acid	1.00	-
	Alpha bisabolol	1.00	-
	Cetyl alcohol	2.50	-
	Compound **16**	0.01	0.01

**Table 2:** Enzymatic inhibition assays.

Compounds	Structure	IC_50_ ± SD (nM)
***6***	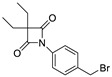	0.64 ± 0.03
***ER 143 (1)***	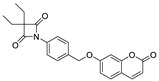	0.42 ± 0.09
***8***	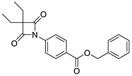	0.96 ± 0.13
***9***	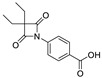	14.71 ± 5.55
***12***	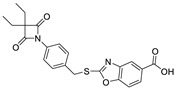	0.38 ± 0.05

**Table 3:** Results of enzymatic inhibition and specificity assays for compounds **16** and **17**. Compound **ER143 (1)** was used as reference.

Compounds	Structure	IC_50_ ± SD (nM)	IC_50_ ± SD (µM)			
		HNE	Trypsin	Chymotrypsin	Urokinase	Kallikrein
***ER143 (1)***	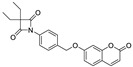	0.42 ± 0.09	>1	>8	>50	>8
***16***	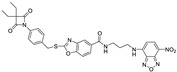	0.85 ± 0.10	>1	>1	>2	>5
***17***	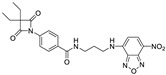	0.64 ± 0.03	>1	>0.5	>2	>3

## Supplementary Materials

The following are available online at https://www.mdpi.com/1999-4923/12/4/358/s1, the characterization of all compounds is presented in the Supplementary Materials.
