# Supplementary material for: Novel and Modified Neutrophil Elastase Inhibitor Loaded in Topical Formulations for Psoriasis Management
Source: Pharmaceutics. 2020 Apr 14;12(4):358. doi: 10.3390/pharmaceutics12040358 (PMC7238052; doi:10.3390/pharmaceutics12040358)
Supplement: Supplementary file 1 [file pharmaceutics-12-00358-s001.pdf]

pharmaceutics

Supplementary Materials: Novel and Modified Neutrophil Elastase Inhibitor Loaded in Topical Formulations for Psoriasis Management

Andreia Nunes, Joana Marto, Lídia Maria Gonçalves, Sandra Simões, Rita Félix, Andreia Ascenso, Francisca Lopes and Helena Margarida Ribeiro

1. Synthesis of 3,3-diethyl-1-(*p*-tolyl)azetidine-2,4-dione **(5)**

To a solution of diethylmalonyl dichloride **(4)** (10.15 mmol, 1.75 mL, 1 eq) in dry DCM (20mL) was added, dropwise, a solution of *p*-toluidine (7.79 mmol, 834.5 mg, 0.77 eq) and triethylamine (30.52 mmol, 4.25 mL, 3 eq) in dry DCM (40 mL). The reaction proceeded in ice bath until was completed (verified by TLC). The solvent was evaporated with formation of a white solid. Cyclohexane (47 mL) was added to the resulting mixture and stirred for 15 min. Then, the solution was filtered, the solvent was evaporated and the product was purified by flash chromatography on silica gel (hexane:ethyl acetate gradient between 9:1 to 5:1) yielding a colorless oil (1.48 g, 82%). **^{1}H NMR** (300 MHz, $CDCl_3$) δ 7.71 (d, *J* = 8.4 Hz, 2H, 2xCH), 7.21 (d, *J* = 8.1Hz, 2H, 2xCH), 2.35 (s, 3H, CH_3), 1.84 (q, *J* = 7.5 Hz, 4H, 2xCH_2), 1.06 (t, *J* = 7.5 Hz, 6H,2xCH_3); **^{13}C NMR** (75 MHz, $CDCl_3$) δ 172.34, 136.75, 131.41, 129.79, 119.23, 72.11, 24.03, 21.26, 9.34; **FTIR** ν_{max} (KBr, cm^{-1}) 2360.87, 2343.51, 1739.79, 1384.89, 1516.05.

2. Synthesis of 1-(4-(bromomethyl)phenyl)-3,3-diethylazetidine-2,4-dione **(6)**

To a solution of **(5)** (5.14 mmol, 1.19 g, 1eq) in dry acetonitrile (45.5 mL), NBS (7.78 mmol, 1.38 g, 1.5 eq) and benzoyl peroxide (0.53 mmol, 127.7 mg, 0.1 eq) were added. The reaction was refluxed (82 °C) under inert atmosphere for 3.5 h. Then, the solvent was evaporated and the product obtained was purified by flash chromatography on silica gel (hexane:ethyl acetate gradient between 9:1 to 5:1) yielding a white solid (1.05 g, 66%), **m.p.** 63-65°C. **^{1}H NMR** (300 MHz, $CDCl_3$) δ 7.84 (d, *J* = 8.5 Hz, 2H, 2xCH_2), 7.44 (d, *J* = 8.5 Hz, 2H, 2xCH_2), 4.48 (s, 2H, CH_2), 1.85 (q, *J* = 7.5 Hz, 4H, 2xCH_2), 1.06 (t, *J* = 7.5 Hz, 6H, 2xCH_3); **^{13}C NMR** (75 MHz, $CDCl_3$) δ 172.20, 136.40, 133.86, 130.14, 119.57, 72.24, 32.76, 24.11, 9.38; **ESI-MS** (+) m/z: 392.1 $[M+H]^+$; **FTIR ν_{max}** (KBr, cm^{-1}) 2360.87, 2343.58, 1743.65, 1516.05, 1379.10, 592.15; **Anal. Calcd**. ($C_{14}H_{16}BrNO_2$): C, 54.21; H, 5.20; N, 4.52. Found: C, 53.95; H, 5.12; N, 3.88%.

3. Synthesis of benzyl 4-aminobenzoate **(7)**

To a solution of 4-aminobenzoic acid (7.32 mmol, 1.00 g, 1 eq) in DMF (40 mL), potassium carbonate (36.41 mmol, 5.03 g, 4.97 eq) and benzyl bromide (8.02 mmol, 954 μL, 1.10 eq) were added. The solution was stirred at room temperature at inert atmosphere for 2 h. Then, the reaction mixture was diluted in ethyl acetate (30 mL) and the product was washed with water (4 × 80 mL). The organic layer was washed with brine, dried with anhydrous Na_2SO_4, filtered and the solvent was evaporated. The obtained product was purified by flash chromatography on silica gel (only hexane, hexane:acetone gradient between 5:1 to 1:1) yielding a yellow solid. (992.8 mg, 60%). m.p. 78-79°C. **^{1}H NMR** (300 MHz, $CDCl_3$) δ 7.89 (d, J = 8.7 Hz, 2H, 2xCH), 7.47 – 7.28 (m, 5H, 5xCH), 6.63 (d, J = 8.8 Hz, 2H, 2xCH), 5.32 (s, 2H, CH_2); **^{13}C NMR** (75 MHz, $CDCl_3$) δ 151.05, 136.75, 131.93, 128.66, 128.18, 128.16, 119.86, 113.95, 66.22; **FTIR** ν_{max} (KBr, cm^{-1}) 3360.00, 2364.73, 2331.94, 1683.86, 1633.71, 1600.92, 1570.06

4. Synthesis of benzyl 4-(3,3-diethyl-2,4-dioxoazetidin-1-yl)benzoate **(8)**

To a solution of diethylmalonyl dichloride **(4)** (5.58 mmol, 960 μL, 1.5 eq) in dry DCM (24 mL) were added, dropwise, a solution of **(7)** (3.62 mmol, 822 mg, 1 eq) and triethylamine (16.7 mmol, 2.33 mL, 4.5 eq) in dry DCM (48 mL). The reaction proceeded in ice bath for 4 h. The solvent was evaporated and cyclohexane (30 mL) was added, and the mixture was stirred for 15 min. Then, the

solution was filtered, the solvent was evaporated and the product purified by flash chromatography on silica gel (hexane:ethyl acetate gradient between 9:1 to 3:1)yielding a white oil (1.13g, 89%). **^{1}H NMR** (300 MHz, $CDCl_3$) δ 8.13 (d, *J* = 8.9Hz, 2H, 2xCH), 7.94 (d, *J* = 8.9Hz, 2H, 2xCH), 7.48 – 7.29 (m, 5H, 5xCH), 5.37 (s, 2H, CH_2), 1.87 (q, *J* = 7.5Hz, 4H, 2xCH_2), 1.07 (t, *J* = 7.5Hz, 6H, 2xCH_3); **^{13}C NMR** (75 MHz, $CDCl_3$) δ172.06, 165.59, 137.63, 135.93, 131.15, 128.76, 128.48, 128.34, 128.06, 118.71, 72.63, 67.04, 24.05, 9.34; **ESI-MS** (+) m/z: 352.0 [M+H]+; **FTIR** ν_{max} (KBr, cm^{-1}) 2360.87, 2343.51, 1743.65, 1716.65.

5. Synthesis of 4-(3,3-diethyl-2,4-dioxoazetidin-1-yl)benzoic acid **(9)**

To solution of **(8)** (0.88mmol, 309.4mg, 1eq) in dry methanol (8.5mL) was added palladium on carbon (10%) (30.9mg), and then the mixture was purged with N_2. The reaction was hydrogenated and proceeded under hydrogen atmosphere for 4 hours. The palladium was filtered in celite and the solvent was evaporated yielding a white solid (221.7mg, 96%), **m.p**. 119-121°C. **^{1}H NMR** (300 MHz, $CDCl_3$) δ 8.17 (d, *J* = 8.9 Hz, 2H,2xCH), 7.99 (d, *J* = 8.9 Hz, 2H, 2xCH), 1.89 (q, *J* = 7.5 Hz, 4H, 2xCH_2), 1.08 (t, *J* = 7.5 Hz, 6H, 2xCH_3); **^{13}C NMR** (75 MHz, $CDCl_3$) δ172.05 , 170.35 , 138.28, 131.7, 127.14, 118.86, 72.75, 24.12, 9.35; **ESI-MS** (+) m/z: 260.1 [M+H]$^+$; **FTIR** ν_{max} (KBr, cm^{-1}) 2362.8, 2345.44, 1743.65, 1683.86, 1606.70; **Anal. Calcd**. ($C_{14}H_{15}NO_4$): C, 64.36; H, 5.79; N, 5.36; O, 24.49%. Found: C, 63.77; H, 5.74; N, 5.31; O, 25.18%.

6. Synthesis of 2-mercaptobenzo[*d*]oxazole-5-carboxylic acid **(11)**

A solution of 3-amino-4-hydroxybenzoic acid **(10)** (4.99mmol, 763.6mg, 1eq) and potassium ethyl xanthate (7.64mmol, 1.22g, 1.5eq) in a mixture of ethanol:water (6:1) was refluxed (80°C) for 5 hours. The reaction mixture was concentrated, under vacuum, and acidified with HCl (1M) until pH 2 with stirring in an ice bath. The product obtained was filtered under vacuum and dried, yielding grey crystals (679.9mg, 70%). This product was used without further purification. **^{1}H NMR** (300 MHz, Acetone-d_6) δ 8.01 (dd, J = 8.5, 1.7Hz, 1H), 7.88 (d, J = 1.1Hz, 1H), 7.54 (d, J = 8.5Hz, 1H)

7. Synthesis of 2-((4-(3,3-diethyl-2,4-dioxoazetidin-1-yl)benzyl)thio)benzo[*d*]oxazole-5-carboxylic acid **(12)**

To a solution of **(11)** (2.36mmol, 460.7mg, 1.4eq) in dry acetone (6mL) was added potassium carbonate (2.14mmol, 295.4mg, 1.3eq), and the resultant solution was stirred for 20 minutes. Then, a solution of **(6)** (1.65mmol, 513.1mg, 1eq) in dry acetone (11mL) was added. The reaction was refluxed (70°C) under inert atmosphere for 6 hours.

The reaction mixture was concentrated, diluted in water and acidified with HCl (1M) up to pH 2 under stirring. The resultant solution was extracted with ethyl acetate and the organic layer was washed with brine, dried with anhydrous Na_2SO_4, filtered and the solvent evaporated. The obtained product was purified by flash chromatography on silica gel (hexane:ethyl acetate gradient between 9:1 to 6:1 and hexane:acetone with increasing polarity between 9:1 to 1:6) yielding a white solid. (321.1mg, 46%), **m.p.** 181-183°C. **^{1}H NMR** (300 MHz, $CDCl_3$) δ 8.38 (d, *J* = 1.1Hz, 1H, CH), 8.08 (dd, *J* = 8.5, 1.7Hz, 1H, CH), 7.83 (d, *J* = 8.5Hz, 2H, 2xCH), 7.54 (d, *J* = 8.6Hz, 2H, 2xCH), 7.50 (d, *J* = 8.6Hz, 1H, CH), 4.56 (s, 2H, CH_2), 1.84 (q, *J* = 7.5Hz, 4H, 2xCH_2), 1.05 (t, *J* = 7.5Hz, 6H, 2xCH_3); **^{13}C NMR** (75 MHz, $CDCl_3$) δ 172.21, 170.31 , 166.23, 155.45, 142.21, 134.52, 130.21, 126.90, 126.05, 121.19, 119.64, 110.02, 72.48, 36.23, 24.10, 9.35; **ESI-MS** (+) m/z: 425.0 [M+H]$^+$; **FTIR** ν_{max} (KBr, cm^{-1}) 2360.87, 2341.58, 1734.01, 1683.86, 1516.05, 1500.62, 1386.82, 678.94; **Anal. Calcd.** ($C_{22}H_{20}N_2O_5S$): C, 62.25; H, 4.75; N, 6.60; S, 7.55; O, 18.85%. Found: C, 62.17; H, 4.81; N, 5.93; S, 7.72; O, 19.37%.

8. Synthesis of *tert*-butyl (3-((7-nitrobenzo[*c*][1,2,5]oxadiazol-4-yl)amino)propyl) carbamate **(14)**

To a solution of tert-butyl (3-aminopropyl) carbamate **(13)** (2.24mmol, 358.1mg, 1eq) in dry methanol (10mL), 4-chloro-7-nitrobenzo[*c*][1,2,5]oxadiazole (2.08mmol, 415.7mg, 0.9eq) in inert atmosphere, was added. The reaction proceeded for 2 hours, at room temperature until completed (by TLC). The product was purified by flash chromatography on silica gel (hexane:ethyl acetate

gradient between 9:1 to 1:6)yielding an orange oil. (272.6mg, 39%). **^{1}H NMR** (300 MHz, $CDCl_3$) δ 8.47 (d, J = 8.6 Hz, 1H, CH), 6.17 (d, J = 8.7 Hz, 1H, CH), 3.60 (q, J = 6.1 Hz, 2H, CH_2), 3.31 (q, J = 6.3Hz, 2H, CH_2), 1.95 – 1.87 (m, 2H, CH_2), (s, 9H, 3xCH_3).

9. Synthesis of *N*^{1}-(7-nitrobenzo[*c*][1,2,5]oxadiazol-4-yl)propane-1,3-diamine **(15)**

To a solution of **(14)** (0.808mmol, 272.6mg, 1eq) in dry DCM (6.2mL) was added TFA (80.8mmol, 6.2mL, 100eq). The resultant solution was stirred at room temperature and inert atmosphere for 30 minutes. A solution of sodium carbonate (1M) was added to the reaction mixture under stirring until pH 8. The resultant solution was extracted with DCM and the organic layer was washed with brine, dried with anhydrous Na_2SO_4, filtered, and the solvent was evaporated yielding an orange solid (57mg, 30%). **^{1}H NMR** (300 MHz, CD_3OD) δ 8.56 (d, J = 8.5 Hz, 1H, CH), 6.42 (d, J = 8.8 Hz, 1H, CH), 2.87 (q, J 4.7 = Hz, 2H, CH_2), 2.03-1.94 (m, 2H, CH_2), 1.02 – 0.92 (m, 2H, CH_2).

10. Synthesis of 2-((4-(3,3-diethyl-2,4-dioxoazetidin-1-yl)benzyl)thio)-*N*-(3-((7-nitrobenzo[*c*][1,2,5]oxadiazol-4-yl)amino)propyl)benzo[*d*]oxazole-5-carboxamide **(16)**

A solution of **(12)** (0.247mmol, 105.0mg, 1eq) in dry DCM (4mL) was maintained at 0°C under inert atmosphere and cool bath. Then, DIPEA (0.259mmol, 45µL, 1.1eq) and TBTU (0.493mmol, 152.6mg, 1.9eq) were added. The reaction proceeded for 3 hours at 0°C and inert atmosphere. When the reaction was completed, compound **(15)** (0.216mmol, 51.4mg, 0.9 eq) and DIPEA (0.259mmol, 45µl, 1.1eq) were added. The reaction proceeded under stirring, cool bath and inert atmosphere for 5 hours. The solvent was evaporated and the product was extracted with ethyl acetate(3x30mL). The organic layer was washed with water and brine, dried with anhydrous Na_2SO_4, filtered, and the solvent was evaporated. The product was purified by flash chromatography on silica gel (hexane:ethyl acetate gradient between 1:1 to 1:5 and only ethyl acetate) yielding an orange solid (74mg, 53%), **m.p**. 109-111°C. **^{1}H NMR** (300 MHz, $CDCl_3$) δ 8.47 (d, *J* = 8.6Hz, 1H, CH), 8.00 (d, *J* = 1.2Hz, 1H, CH), 7.82 (d, *J* = 8.5Hz, 2H, 2xCH), 7.78 (dd, *J* = 8.5, 1.7 Hz, 1H, CH), 7.53 (d, *J* = 8.5Hz, 2H, 2xCH_2), 7.49 (d, *J* = 8.5Hz, 1H, CH), 6.19 (d, *J* = 8.6Hz, 1H, CH), 4.54 (s, 2H, CH_2), 3.72-3.66 (m, 4H, 2xCH_2), 2.10 – 2.02 (m, 2H, CH_2), 1.84 (q, *J* = 7.5Hz, 4H, 2xCH_2), 1.05 (t, *J* = 7.5Hz, 2xCH_3); **^{13}C NMR** (75 MHz, $CDCl_3$) δ 172.23, 166.21, 162.60, 154.25, 144.75, 136.58, 134.60, 133.60, 130.59, 130.21, 123.88 , 122.40, 120.46, 119.61, 117.29, 110.32, 83.04, 72.44, 40.23, 37.31, 36.24, 28.63, 24.09, 9.02; **ESI-MS** (+) m/z: 642.3 $[M+H]^+$; **FTIR** ν_{max} (KBr, cm^{-1}) 3325.28, 2303.01, 1735.93, 1581.63, 1496.76, 1296.16, 1261.45, 1132.21; **Anal. Calcd**. ($C_{31}H_{29}N_7O_7S$): C, 57.85; H, 4.54; N, 15.23; S, 4.98; O, 17.84%. Found: C, 58.55; H, 5.35 N, 12.69; S, 5.01; O, 18.4%

11. Synthesis of 4-(3,3-diethyl-2,4-dioxoazetidin-1-yl)-*N*-(3-((7-nitrobenzo[*c*][1,2,5] oxadiazol-4-yl)amino)propyl)benzamide **(17)**

A solution of **(9)** (0.294mmol, 76.9mg, 1eq) in dry DCM (4mL) was maintained at 0°C under inert atmosphere and cool bath. Then, DIPEA (0.324mmol, 56.4µL, 1.1eq) and TBTU (0.572mmol, 217.6mg, 1.9eq) were added. The reaction proceeded under stirring for 30 minutes. Compound **(15)** (0.279mmol, 66.3mg, 0.95eq) and DIPEA (0.32 mmol, 56.4µl, 1.1eq) were added to the mixture and the reaction was stirred for 5 hours. The solvent was evaporated and the product was extracted with ethyl acetate(3x30mL). Then the organic layer was washed with water and brine, dried with anhydrous Na_2SO_4, filtered, and the solvent was evaporated. The product was purified by flash chromatography on silica gel (hexane:ethyl acetate gradient between 9:1 to 1:9 and acetone and methanol:acetone 1:9) yielding an orange solid (85mg, 63%), **m.p.** 83-84°C. **^{1}H NMR** (300 MHz, $CDCl_3$) δ 8.47 (d, *J* = 8.7Hz, 1H, CH), 7.97 (d, *J* = 8.9Hz, 2H, 2xCH_2), 7.88 (d, *J* = 8.9Hz, 2H, 2xCH_2), 6.19 (d, *J* = 8.7Hz, 1H, CH), 3.70-3.64 (m, 4H, 2xCH_2), 2.09 – 2.00 (m, 2H, CH_2), 1.89 (q, *J* = 7.5Hz, 4H, 2xCH_2), 1.08 (t, *J* = 7.5Hz, 6H, 2xCH_3); **^{13}C NMR** (75 MHz, $CDCl_3$) δ 172.10, 168.01, 144.48, 136.87, 136.73, 131.41, 128.41, 123.43, 119.08, 98.01, 72.70, 39.98, 37.15, 29.06, 24.06, 9.37; **ESI-MS** (+) m/z: 479.2 $[M+H]^+$; **FTIR** ν_{max} (KBr, cm^{-1}) 2366.66, 2345.44, 1737.86, 1568.13, 1504.48, 1307.74, 1296.16, 1269.16, 1124.50; **Anal. Calcd**. ($C_{23}H_{24}N_6O_6$): C, 57.50; H, 5.03; N, 17.49%. Found: C, 57.01; H, 5.75; N, 14.23%
